# Adsorption Properties of Metal Atom (Co, V, W, Zr)-Modified MoTe_2_ for CO, CH_3_CHO, and C_6_H_6_ Gases: A DFT Study

**DOI:** 10.3390/molecules29215086

**Published:** 2024-10-28

**Authors:** Weizhong Xiao, Zixuan Wang, Yingang Gui

**Affiliations:** 1 College of Energy Engineering, Huanghuai University, Zhumadian 463000, China; xwz820813@outlook.com; 2 School of Intelligent Manufacturing, Huanghuai University, Zhumadian 463000, China; 3 College of Engineering and Technology, Southwest University, Chongqing 400715, China; yinganggui@swu.edu.cn

**Keywords:** metal atom modification, MoTe_2_, hazardous gases, DFT, gas detection

## Abstract

This study investigates the adsorption characteristics of the pristine MoTe_2_ monolayer and the metal atom (Co, V, W, Zr)-modified MoTe_2_ monolayer on the hazardous gases CO, CH_3_CHO, and C_6_H_6_ based on the density functional theory. The adsorption mechanism was studied from the perspectives of molecular density differences, band structures, molecular orbitals, and the density of states. Research analysis showed that the changes in conductivity caused by the adsorption of different gases on the substrate were significantly different, which can be used to prepare gas sensing materials with selective sensitivity for CO, CH_3_CHO, and C_6_H_6_. This study lays a reliable theoretical foundation for the gas sensing analysis of toxic and hazardous gases using metal atom-modified MoTe_2_ materials.

## 1. Introduction

Carbon monoxide (CO) is a colorless and odorless gas mainly derived from the incomplete combustion of fuels, such as gasoline, diesel, coal, etc. [1,2,3]. Carbon monoxide mainly comes from the exhaust and the incomplete combustion of an engine, especially when the combustion engine keeps turning on under a standby state. Long term exposure to CO may cause symptoms such as headache, dizziness, drowsiness, and even death [4,5,6]. Acetaldehyde (CH_3_CHO) is an organic compound with a pungent odor. CH_3_CHO is a common decomposition product of plastic parts, rubber parts, and other interior materials, while benzene (C_6_H_6_) often releases from materials such as adhesives and leather in cars [7,8,9,10,11]. It is worth noting that both CH_3_CHO and C_6_H_6_ can cause serious damage to the human body at high concentrations. The harmful substances in the interior decoration materials release a lot of volatile organic pollutants [12,13,14]. In order to maintain human health and safety, it is necessary to detect and adsorb CO, CH_3_CHO and C_6_H_6_ inside cars.

Since the emergence of graphene, two-dimensional materials have received attention from researchers in the field of gas sensing. Abbasi et al. found that the interaction between Al- or Si-atom-modified MoS_2_ and gas molecules is stronger than that between the pristine MoS_2_ and gas molecules, and can sensitively detect CO and NO in the environment [15]. In addition, they also constructed undoped and N-doped multi-component heterostructures, TiO_2_/WSe_2_ [16] and TiO_2_/MoS_2_ [17,18], to improve the gas adsorption of the material. Layered transition metal disulfides (TMDs), one of the materials with the highest degree of two-dimensional transformation, overcome the shortcomings of graphene’s zero bandgap characteristics and demonstrate enormous application potential. Each layer is composed of an alternating arrangement of transition metal atoms (Mo, Ti, W, etc.) and sulfur group element atoms (S, Se, Te, etc.), which are connected to each other through van der Waals force interactions [19,20,21]. This structure enables TMDs to have rich electronic structural states, such as topological insulating states, semi-conductive states, semi-metallic states, and superconducting states [22,23]. In addition, TMDs also have excellent optical [24,25,26] and structural properties [27,28], making them an ideal two-dimensional platform for studying various potential applications. Among the TMDs studied so far, MoTe_2_ is considered a potential efficient sensing material due to its fast response [29]. It is worth mentioning that the homojunction efficiency of MoTe_2_ reaches 10–50 times that of silicon, and electrons can move quickly [30,31]. According to the topological structure, MoTe_2_ shows typical semiconductor characteristics [32,33,34]. A large amount of theoretical and experimental research has proven that the pristine MoTe_2_ has a high sensing ability as an efficient gas detection device [35,36,37,38].

The surface modification of transition metals has been experimentally proven to be an effective way to improve the gas sensing properties of materials [39,40,41]. Wang et al. found that a MoTe_2_ monolayer modified with Ni atoms significantly improved their adsorption capacity for SF_6_ decomposition gases [42]. Lin et al. studied the adsorption behavior of Ag, Pd, and Rh atoms with different modification methods, and found that both the conductivity and chemical activity were improved after the metal atom modifications [43]. However, there is still limited research on the metal-element-modified MoTe_2_. Han et al. used redox V-metal organic framework materials to adsorb NO_2_ gas, and experimental results showed a high NO_2_ absorption rate and excellent adsorption reversibility [44]. Zirconium based materials, such as zirconium-based metal organic frameworks (Zr-MOFs), are widely studied for gas adsorption and separation due to their high specific surface area and adjustable pore structure [45]. Related reports also indicate that W and Co atom modifications be used to prepare high-performance gas sensing materials, and the introduction of TM doping shows a significant enhancement of adsorption selectivity [46,47,48,49]. Therefore, this study explored the electronic properties of Co, V, W, and Zr-modified MoTe_2_ monolayers (abbreviated as Metal-MoTe_2_), and investigated its adsorption on the hazardous gases CO, CH_3_CHO, and C_6_H_6_ through geometric optimization, charge density difference (CDD), band structure, the density of states, molecular orbitals, and desorption time analysis.

## 2. Results and Discussion

### 2.1. Structural Optimization of Gases, MoTe_2_, and Metal-MoTe_2_

**Figure 1:**
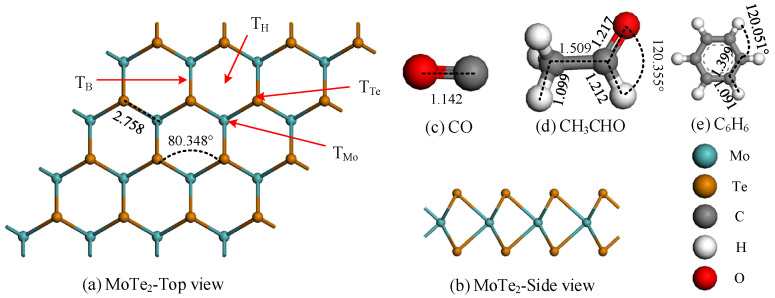
Optimization Structure of CO, CH_3_CHO, C_6_H_6_, and the MoTe_2_ monolayer, distance in Å.

Figure 1a,b show the optimized geometrical structure of the pristine MoTe_2_ monolayer. The Mo and Te atoms were arranged alternately, with Mo atoms located in the middle of the monolayer, forming hexagonal rings. The bond length between the Mo atom and Te atom was 2.758 Å, and the angle formed by Te-Mo-Te was 80.348°. Among them, there were four typical adsorption sites on the MoTe_2_ monolayer: T_H_ (middle of the hexagon), T_Te_ (above the Te atom), T_Mo_ (above the Mo atom), and T_B_ (above the bond connecting the Te and Mo atoms). In Figure 1c, the covalent bond length between the C and O atoms of the CO molecule was 1.142 Å, forming three bonds. The bond length of the CH_3_CHO molecule is shown in Figure 1d, and the angle between O-C-H was 120.355°. The six carbon atoms of C_6_H_6_ formed a cyclic closed chain, with a C-C bond length of 1.399 Å. Each C atom was surrounded by an H atom, with a length of 1.091 Å. In addition, a comparison of the structural parameters (MoTe_2_, CO, CH_3_CHO, and C_6_H_6_) were obtained from our theoretical calculations and experimental tests from references, which are listed in Table S1.

**Figure 2:**
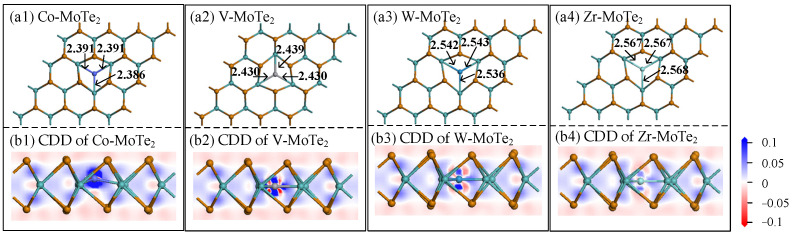
The modification structure and charge density differences (CDD) of the Metal-MoTe_2_.

The energy released when a MoTe_2_ monolayer binds to metal atoms (Co, V, W, or Zr) is defined as the binding energy (*E*_b_). The larger the absolute value of *E*_b_, the more stable the structure is. In order to obtain the most stable configuration, the *E*_b_ and the nearest atomic adsorption distance were comprehensively considered. The optimal structures of the Metal-MoTe_2_ monolayer are shown in Figure 2(a1–a4), where it can be seen that the Co (or V, W, Zr) atom located to the middle of the hexagon, indirectly being surrounded by the three Mo atoms. And, the edges of the hexagon where the metal atom was located underwent certain deformations compared to that before binding, indicating that corresponding chemical reactions occurred during the modification process. The *E*_b_ between the Co atom and MoTe_2_ monolayer was −7.264 eV, indicating that the binding between an atom and the substrate was an exothermic process and very tight. At the same time, the Co atom, as an electron acceptor, obtained a charge of 0.338 *e*, and the large charge transfer and absolute adsorption energy indicated that intense chemical reactions occurred during the binding process.

The charge density difference (CDD) and band structure of the Metal-MoTe_2_ monolayer were also carefully analyzed. Figure 2(b1–b4) show the CDD of the Metal-MoTe_2_ monolayer. The blue color represents an increase in the charge density in the region, while the red color represents the opposite. The darker the color, the more the charge density in that area increased or decreased. From Figure 2(b1), it can be seen that the Co atom was tightly adhered to a deeper blue color and there was more electron aggregation in the bonding area, making the modification very stable. From Figure 2(b2), it can also be seen that strong charge transfer and redistribution occurred near the V atom, which was consistent with the phenomenon of a large *E*_b_ (−9.144 eV) and a distinct charge transfer (the MoTe_2_ monolayer lost 0.230 *e*). The chemical reactions between the W/Zr atom and the substrate were relatively weak compared to the first two. The W atom and Zr atom obtained charges of 0.171 *e* and 0.216 *e* from the MoTe_2_ monolayer with *E*_b_ values of −5.624 eV and −3.283 eV, respectively.

**Figure 3:**
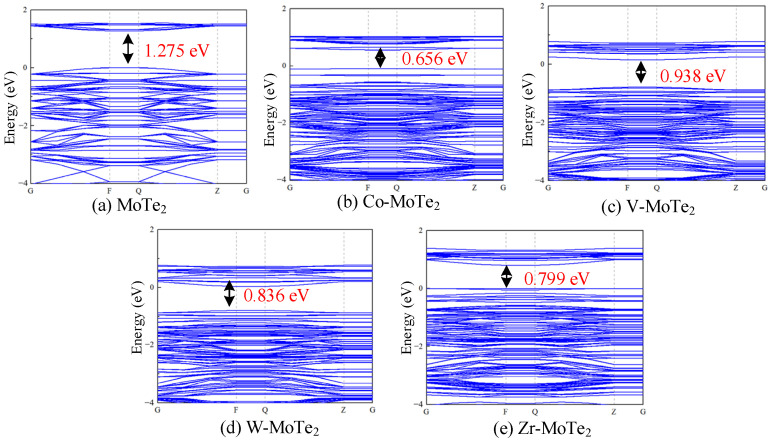
Energy band structure diagrams of the pristine MoTe_2_ and Metal-MoTe_2_.

By observing the band structure shown in Figure 3 and comparing the relative positions and widths of the valence and conduction bands, the conductivity of the material was determined. As shown in Figure 3, the energy gap of the pristine MoTe_2_ was 1.275 eV, while the energy gap decreased to varying degrees after modification with the four different atoms. The energy gaps arranged in order from small to large were Co-MoTe_2_ < Zr-MoTe_2_ < W-MoTe_2_ < V-MoTe_2_ < MoTe_2_. The reduction in the energy gap made it easier for the electrons to jump between the valence and conduction bands, and atomic modification had a positive impact on the conductivity of the material.

### 2.2. Adsorption Analysis of CO, CH_3_CHO, C_6_H_6_ on the Metal-MoTe_2_

#### 2.2.1. Structural Analysis

**Figure 4:**
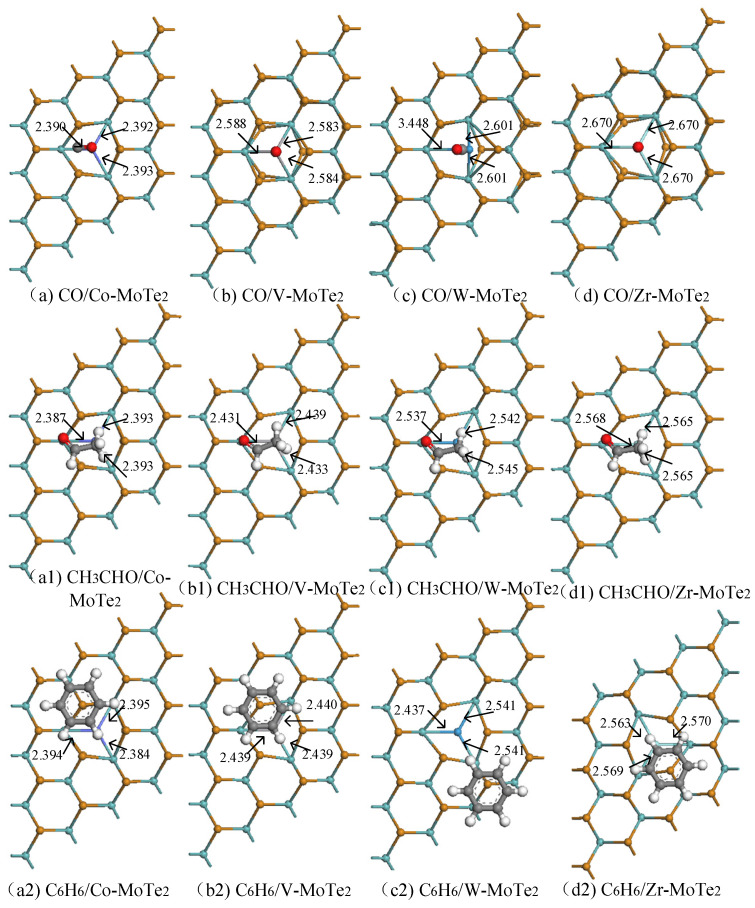
The adsorption structures of the Metal-MoTe_2_.

In order to directly perceive the adsorption behavior of the Metal-MoTe_2_ monolayer on CO, CH_3_CHO, and C_6_H_6_ gases, the related adsorption structures were plotted and the relevant parameters were summarized, as shown in Figure 4 and Table 1, respectively. In addition, the adsorption parameters of the pristine MoTe_2_ monolayer for these three hazardous gases are also summarized, which were used to effectively compare and analyze the relevant parameters of the pristine system and the modification system. Adsorption energy (*E*_ads_) refers to the interaction energy between the adsorbate and the adsorbent during the adsorption process. In this work, the magnitude of *E*_ads_ is defined as the sum of the energy of the gas molecules before adsorption and the energy of the substrate minus the energy of the substrate after adsorption of the gas. A negative *E*_ads_ indicates that this is an exothermic reaction, and that the adsorption process can occur spontaneously. Previous studies have shown that the addition of van der Waals interactions during adsorption increases the adsorption energy [16,50,51]. This article corrected for the influence of van der Waals interactions, but the calculation results showed that the adsorption energy was relatively large, so the influence of van der Waals interactions on the adsorption energy was relatively small. Overall analysis showed that when the Co-MoTe_2_ adsorbed three gases, the bond length of Co-Mo changed very little, which may be related to the higher binding energy between the Co atom and the MoTe_2_ monolayer. Compared with the pristine Co atom, the shortest atomic spacing for adsorbing CO gas after modification was decreased, while *E*_ads_ (−0.167 eV) decreased to about one-twentieth of the original adsorption energy, significantly reducing the adsorption energy. A smaller adsorption energy is beneficial for the desorption of small gas molecules, improving the reuse rate of the substrate. The adsorption energies of Co-MoTe_2_ for CH_3_CHO and C_6_H_6_ were −0.392 eV and −0.667 eV, respectively, with a slight increase in the shortest atomic spacing comparing to that before modification. In the V-MoTe_2_ system, the adsorption distance of CO was 54.7% of the original, and the *E*_ads_ of CH_3_CHO changed the most, decreasing to one-ninth of the original. The trend of changes in the CO adsorption parameters for the W-MoTe_2_ system and the Zr-MoTe_2_ system was the same. The adsorption distance of CO decreased to about half of the original, and the CO molecules were almost vertically downwards, with the closest distance between the C atom and the metal atom. The shortest atomic distance between W-MoTe_2_ and Zr-MoTe_2_ for CH_3_CHO was between the H and Te atoms, and the distances were increased, with *E*_ads_ of −0.386 eV and −0.439 eV, respectively. After W-MoTe_2_ and Zr-MoTe_2_ adsorbed C_6_H_6_, both of the C_6_H_6_ molecules were parallel to the substrate, and the *E*_ads_ decreased compared to the original. From this, it can be seen that the adsorption energy of the modification system for gases decreases, and the adsorption ability of the modified atoms between the substrate and the CO, CH_3_CHO, C_6_H_6_ gases weakens.

#### 2.2.2. Electronic Analysis

**Figure 5:**
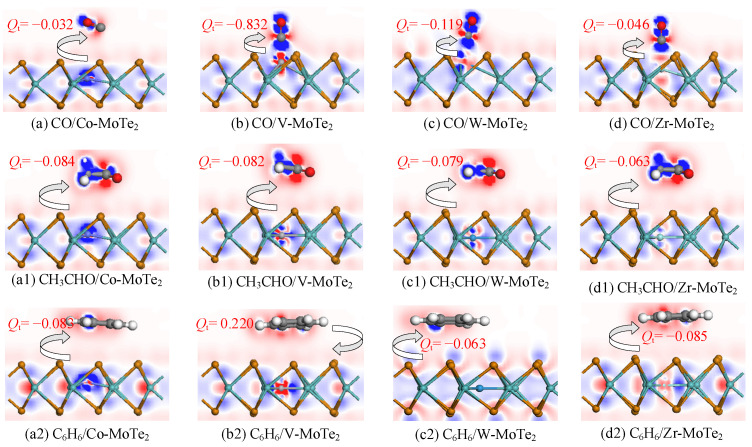
The CDD of gas molecule adsorption on the Metal-MoTe_2_.

**Figure 6:**
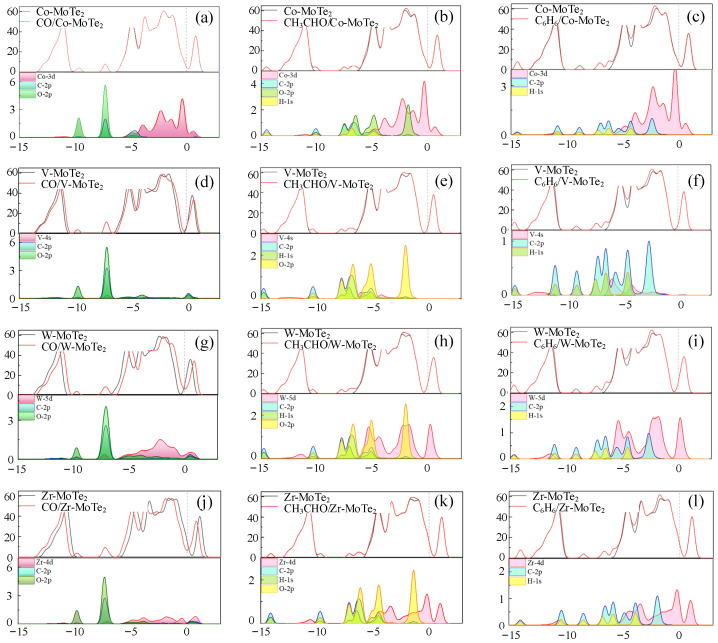
The TDOS and PDOS on the Metal-MoTe_2_ before and after gas molecule adsorption. (**a**,**d**,**g**,**j**) CO adsorption. (**b**,**e**,**h**,**k**) CH_3_CHO adsorption. (**c**,**f**,**i**,**l**) C_6_H_6_ adsorption.

To gain a detailed understanding and study of the behavior and properties of the electrons during the adsorption process, Figure 5 and Figure 6 depict the charge density difference (CDD) and the density of states (DOS) of the gas adsorption on the Metal-MoTe_2_ monolayers. For the adsorption calculation, the Hirshfeld method is used to calculate the charge transfer during the adsorption process, which can better reflect the actual distribution of electron clouds [52]. Using the Hirshfeld charge analysis method, *Q*_t_ defines the amount of charge transferred from the gas molecules to the surface. *Q*_t_ < 0 indicates the transfer of electrons from the surface to the gas molecules.

From Figure 5(a,a1,a2), it can be seen that the Co-MoTe_2_ monolayer lost electrons as a donor when adsorbing CO, CH_3_CHO and C_6_H_6_. The strong blue color near the Co atom indicated its significant influence on gas adsorption. In the CO/Co-MoTe_2_ system, although the C atom lost electrons, the oxygen atom gained more electrons, thus CO overall gained electrons. In Figure 5(b,b1,b2), the V atom appeared with red around it, and the electron density decreased during the adsorption process. The charge transfer amount was the highest in the process of the V-MoTe_2_ monolayer adsorption of CO, with a charge transfer amount of 0.832 *e*. It is worth noting that when V-MoTe_2_ adsorbed C_6_H_6_, although both the V and Te atoms lost electrons, the electron density near the Mo atoms increased, resulting in a transfer of 0.22 *e* electrons from the gas to the substrate. When using four different substrates to adsorb CH_3_CHO, the C atom in the aldehyde group of CH_3_CHO showed a strong red color, indicating that the aldehyde group in acetaldehyde is prone to losing electrons during the adsorption process. In the Zr-MoTe_2_ system, the charge transfer amount was relatively small, and as shown in Table 1, the adsorption distance was also large, indicating that the adsorption reaction may be a chemical reaction.

From Figure 6, gas adsorption affected the electronic properties of the substrate and the distribution of electrons at different energy states. In Figure 6a–c, the partial density of states (PDOS) distribution of the Co-3*d* orbital at the Fermi level was the largest, indicating that atomic modification had a significant impact on the conductivity of the material. After CO adsorption, the TDOS remained almost unchanged and exhibited weak adsorption. After adsorption of CH_3_CHO, although the TDOS near the Fermi level remained almost unchanged, three new peaks appeared in the range of −10–−6 eV. Observing the PDOS graph, it can be seen that the new peaks were likely caused by the superposition of the gases’ molecular density of states. Unlike the previous two gases, after adsorbing C_6_H_6_, there was a significant increase in the TDOS in the valence band within the range of −6 eV to −2 eV, indicating that adsorption affected the electronic orbitals of the crystal. In the V-MoTe_2_ system, as shown in Figure 6d, the TDOS shifted to the right, the TDOS near the Fermi level slightly increased, the gap between the valence band and the conduction band decreased, and the conductivity slightly increased. The charge transfer amount of CH_3_CHO adsorbed in the V-MoTe_2_ process was very small, which corresponded to the TDOS that remained almost unchanged at the Fermi level. The slight increase in the TDOS near −2 eV and within the range of −10 eV to −6 eV was mainly caused by the O-2*p* and H-1*s* orbitals of CH_3_CHO. In Figure 6g, after CO adsorption on W-MoTe_2_, the TDOS curve shifted to the right, with continuous valence, conduction bands, and continuous energy level distribution, which improved the conductivity of the system. In Figure 6h, the peaks of the W-5*d*, O-2*p*, and H-1*s* orbitals overlapped near −2 eV and within the range of −10 eV to −6 eV, indicating severe hybridization of the atomic orbitals and strong interatomic interactions. From Figure 6i, it can be seen that new peaks appeared in the TDOS near −14 eV, −9 eV, and −7 eV. At the same time, according to the PDOS, there was a significant hybridization of the W-3*d* orbital and the C-2*p* orbital near −6 eV and −4.5 eV. It is speculated that the appearance of the new peaks may be caused by electron redistribution in the substrate. After Zr-MoTe_2_ adsorbed CO, the TDOS shifted to the left, and the TDOS near the Fermi level decreased. However, the valence and conduction bands were continuous, and electrons could freely transition between the different energy levels. Within the range of −5 eV to 0 eV, weak hybridization occurred between the Zr-4*d* orbital and the C-2*p* orbital. In Figure 6l, orbital hybridization was evident near −10.5 eV, −8 eV, −6 eV, and −4 eV. Therefore, the TDOS and conductivity slightly increased near the valence band.

#### 2.2.3. Molecular Orbital Analysis

This work applied molecular orbital theory to analyze the changes in the electrical conductivity of materials after adsorbing gases. HOMO and LUMO refer to the highest occupied molecular orbital and the lowest unoccupied molecular orbital, respectively, and the absolute difference between the two is the size of the energy gap (*E*_g_). The smaller the energy level difference, the easier it is for molecules to be excited, that is, the better the conductivity of the material. The electrons on the HOMO orbital are the easiest to remove, while the LUMO orbital is the easiest to obtain electrons.

**Figure 7:**
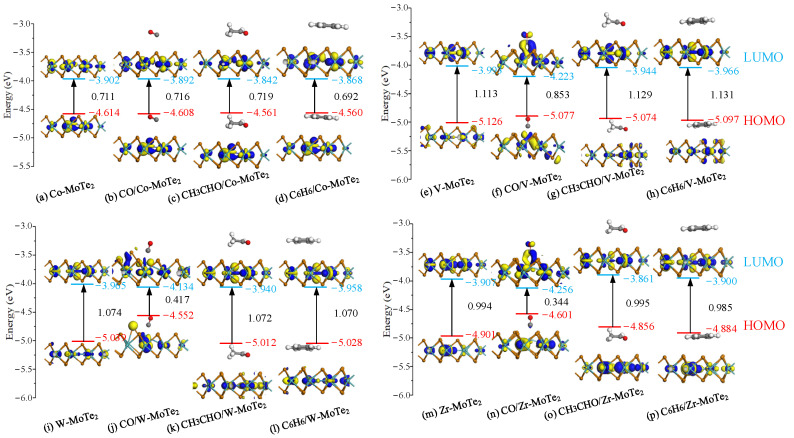
HOMO and LUMO on the Metal-MoTe_2_ before and after gas molecule adsorption.

Figure 7 shows the HOMO and LUMO on the Metal-MoTe_2_ before and after gas molecule adsorption. As shown in Figure 7, LUMO and HOMO mainly distributed around the metal atoms and the Mo atoms. When the Metal-MoTe_2_ monolayer adsorbed CH_3_CHO and C_6_H_6_, there were no HOMO and LUMO near the gas, which corresponded to the small charge transfer between the substrate and the gas shown in Figure 5. In the Co-MoTe_2_ system, after the adsorption of CO, CH_3_CHO, and C_6_H_6_, the band gap changed little, with a variation of about 1%. In the V-MoTe_2_ system, after adsorbing CO, the energy gap decreased by 23.4% and the conductivity increased. However, after adsorbing CH_3_CHO and C_6_H_6_, the *E*_g_ increased by 1.4% and 1.6%, respectively. For W-MoTe_2_, the *E*_g_ decreased after adsorbing all three gases, but the *E*_g_ changed the most after adsorbing CO, from 1.074 eV to 0.417 eV, with a change rate of 61.2%. The *E*_g_ values of the other two gases only slightly decreased. After Zr-MoTe_2_ adsorbed CH_3_CHO, the *E*_g_ value increased by 0.1%, while after adsorbing CO and C_6_H_6_, the *E*_g_ decreased by 66.4% and 1%, respectively. The reduction in *E*_g_ made it easier for electrons to jump between the valence band and conduction band, thereby improving the conductivity of the material.

### 2.3. Comparison of the Adsorption Performance in Different Systems

**Figure 8:**
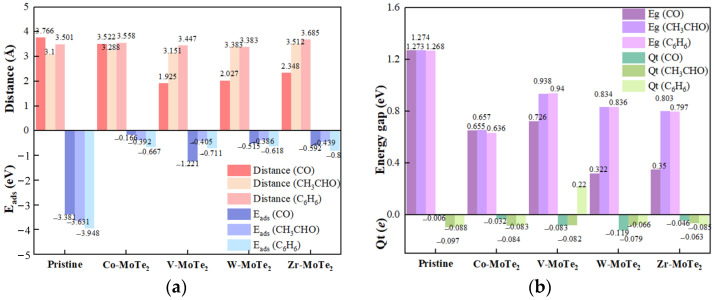
Comparisons of the band gap (**a**) and adsorption energy (**b**) of gas adsorption on the MoTe_2_ and Metal-MoTe_2_.

To better investigate the effect of the modified metal atoms (Co, V, W, Zr) on the gas adsorption performance of the pristine MoTe_2_ monolayer, Figure 8 shows the adsorption distance, *E*_ads_, *E*_g_, and *Q*_t_ of the pristine MoTe_2_ and the Metal-MoTe_2_ adsorbed gas. Firstly, according to Figure 8, the adsorption energy of the substrate for the three gases significantly reduced after modification. CO molecules are inorganic small gas molecules, while CH_3_CHO and C_6_H_6_ are organic small molecules. Generally speaking, adsorbents with lower adsorption energy typically have a larger specific surface area and more adsorption sites, which enables them to more effectively adsorb small gas molecules [52,53]. Secondly, the shortest atomic distance and *Q*_t_ of CO adsorbed after modification significantly decreased and increased compared with that before adsorption, while the shortest atomic distance and *Q*_t_ of CH_3_CHO and C_6_H_6_ did not change significantly, indicating that the modification operation may not have significantly changed the adsorption capacity and charge transfer capacity of the substrate. Then, by modification with metal atoms, the decrease in *E*_g_ indicated that the energy required for electronic transitions was reduced, and the conductivity of the material was improved. Finally, Table 2 lists the adsorption characteristics and parameters of some of the two-dimensional gas sensitive materials for CO, CH_3_CHO, and C_6_H_6_, which was helpful for performance comparison. From the comparison in Table 2, it was found that although the Metal-MoTe_2_ monolayer had a larger shortest atomic distance for CO, CH_3_CHO, and C_6_H_6_ gases, its adsorption energy was relatively moderate. Overall, the Metal-MoTe_2_ monolayer was suitable for monitoring the concentration of hazardous gases such as CO, CH_3_CHO, and C_6_H_6_, or for adsorbing gases.

### 2.4. Desorption Performance Analysis

**Figure 9:**
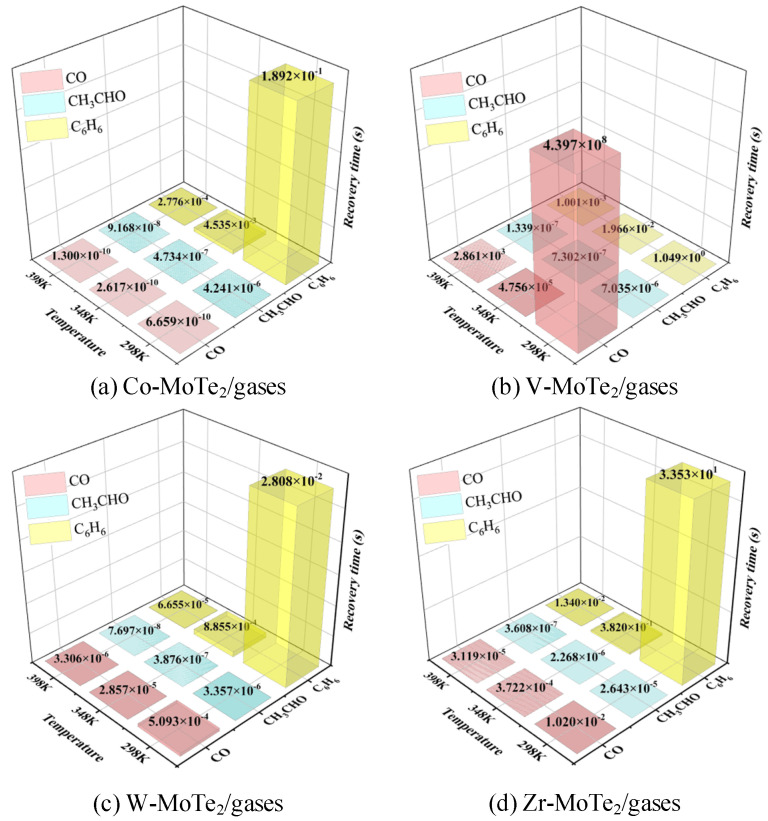
The recovery time of the Metal-MoTe_2_ on CO, CH_3_CHO, and C_6_H_6_ at 273 K, 298 K, and 318 K.

Good desorption performance can not only help improve the sensitivity and selectivity of gas sensing materials, but also extend the service life and improve their anti-interference ability of gas sensing materials. Recovery time is an important parameter reflecting parsing performance, usually defined in Equation (1) [58,59]:

(1) $$\tau =\nu_{0}^{-1}e^{\left(-E_{\mathrm{ads}}/K_{B}T\right)}$$

where *v*_0_ is the trial frequency and *K*_B_ is the Boltzmann constant. It can be inferred that the temperature and *E*_ads_ have a significant impact on the recovery time. Figure 9 shows the recovery times of the Metal-MoTe_2_ for CO, CH_3_CHO, and C_6_H_6_ at 298 K, 348 K, and 398 K. From Figure 9a,c,d, it can be seen that the Co-MoTe_2_ monolayer and the W-MoTe_2_ monolayer had a short recovery time for hazardous gases in the range of 298 K to 398 K, which could quickly adapt to environmental changes and provide more real-time data feedback. This is particularly important for applications that require rapid response. The gas recovery time of the Zr-MoTe_2_ monolayer was also acceptable. Co-MoTe_2_, W-MoTe_2_, and Zr-MoTe_2_ are all ideal gas sensing materials for CO, CH_3_CHO, and C_6_H_6_ gases with appropriate adsorption energies for gases. On the contrary, the V-MoTe_2_ monolayer had a longer recovery time for the CO gas, which was not conducive to improving the material’s reuse rate. Therefore, V-MoTe_2_ is suitable as a material for monitoring the concentration of CH_3_CHO and C_6_H_6_, and can serve as an adsorbent for CO molecules.

## 3. Methods

All calculations in this study were performed based on the density functional theory using Dmol^3^ 8 (Bernard Delley, Switzerland). After consulting simulation literature on MoTe_2_ [38,60,61], the Monkhorst–Pack K point grid was set to 7 × 7 × 1, and the generalized gradient approximation (GGA) PBE function to calculate the exchange correlation energy was used [62]. In all calculations, the convergence accuracy of the electron self-consistent field and the ion self-consistent field were 1.0 × 10^−6^ Ha and 1.0 × 10^−5^ Ha, respectively. The calculation parameters for the maximum displacement and the maximum force were set to 0.005 Å and 0.002 Ha/Å, respectively. In order to avoid the influence between the adjacent layers, a 4 × 4 × 1 supercell with a vacuum plate exceeding 20 Å was established [63]. In order to improve the accuracy and efficiency of the calculations and predict the magnetic properties of the materials, spin polarization was also used in the calculations [64].

## 4. Conclusions

This study applied the density functional theory to study the adsorption of the pristine MoTe_2_ monolayer and Co, V, W, Zr-modified MoTe_2_ monolayers for the hazardous gases CO, CH_3_CHO, and C_6_H_6_ from the perspectives of structural optimization, adsorption structure analysis, electronic analysis, molecular orbital analysis, and desorption performance analysis. On the one hand, the structural optimization results showed that the Co, V, W, Zr-modified MoTe_2_ form stable structures with large bind energy values of −7.264 eV, −9.144 eV, −5.624 eV, and −3.283 eV, respectively. On the other hand, the modified metal atom (Co, V, W, Zr) enhanced the conductivity of the MoTe_2_ monolayer, and acted as active adsorption sites for the gas molecules. The adsorption performance of the different gases on the pristine MoTe_2_ monolayer and Co, V, W, Zr-modified MoTe_2_ monolayers were compared. The adsorption energy decreased after metal atom (Co, V, W, Zr) modification, which significantly reduced the gas desorption time. Based on DOS and the desorption performance analysis, Co, V, W, Zr-modified MoTe_2_ monolayers showed different conductivity variation upon CO, CH_3_CHO, and C_6_H_6_ adsorption, which plays a theoretical basis for preparing gas sensing materials with good sensitivity selectivity.

## Figures and Tables

**Table 1:** The adsorption structure, adsorption distance, and *E*_ads_ of the gases on Metal-MoTe_2_.

System	Structure	Distance (Å)	*E*_ads_ (eV)
CO/Co-MoTe_2_	Figure 4a	C-Te:3.522	−0.167
CH_3_CHO/Co-MoTe_2_	Figure 4(a1)	C-Te:3.288	−0.392
C_6_H_6_/Co-MoTe_2_	Figure 4(a2)	H-Te:3.558	−0.667
CO/V-MoTe_2_	Figure 4b	C-V:1.925	−1.221
CH_3_CHO/V-MoTe_2_	Figure 4(b1)	H-Te:3.151	−0.405
C_6_H_6_/V-MoTe_2_	Figure 4(b2)	H-Te:3.447	−0.711
CO/W-MoTe_2_	Figure 4c	C-W:2.027	−0.515
CH_3_CHO/W-MoTe_2_	Figure 4(c1)	H-Te:3.383	−0.386
C_6_H_6_/W-MoTe_2_	Figure 4(c2)	C-Te:3.708	−0.618
CO/Zr-MoTe_2_	Figure 4d	C-Zr:2.348	−0.592
CH_3_CHO/Zr-MoTe_2_	Figure 4(d1)	H-Te:3.512	−0.439
C_6_H_6_/Zr-MoTe_2_	Figure 4(d2)	H-Te:3.685	−0.800

**Table 2:** The adsorption characteristics of different materials for detecting CO, CH_3_CHO and C_6_H_6_.

Gas	Material	*E*_ads_ (eV)	*Q*_t_ (*e*)	Distance (Å)	Reference
CO	Ni-MoTe_2_	−2.23	0.729	1.750	[6]
	MoO_3_	−0.1	0.1	2.4	[54]
	Co-MoTe_2_	−0.167	−0.032	3.522	This work
	V-MoTe_2_	−1.221	−0.832	1.925	
	W-MoTe_2_	−0.515	−0.119	2.027	
	Zr-MoTe_2_	−0.592	−0.046	2.348	
CH_3_CHO	Fe-MoS_2_	−1.77	−0.179	1.865	[55]
	Al-Graphene	−3.183	−0.208	/	[56]
	Co-MoTe_2_	−0.392	−0.084	3.288	This work
	V-MoTe_2_	−0.405	−0.082	3.151	
	W-MoTe_2_	−0.386	−0.079	3.383	
	Zr-MoTe_2_	−0.439	−0.063	3.512	
C_6_H_6_	Fe-MoS_2_	−1.88	0.094	1.246	[55]
	Pd-GeSe	−0.51	0.050	/	[57]
	Co-MoTe_2_	−0.667	−0.083	3.558	This work
	V-MoTe_2_	−0.711	0.220	3.447	
	W-MoTe_2_	−0.618	−0.063	3.708	
	Zr-MoTe_2_	−0.800	−0.085	3.685	

## Data Availability

Data are contained within the article.

## Supplementary Materials

The following supporting information can be downloaded at: https://www.mdpi.com/article/10.3390/molecules29215086/s1, Table S1: The structure of MoTe2, CO, CH_3_CHO, and C_6_H_6_ gases. References [65,66,67,68] are cited in the Supplementary Materials.
