# Supplementary material for: Adsorption Properties of Metal Atom (Co, V, W, Zr)-Modified MoTe2 for CO, CH3CHO, and C6H6 Gases: A DFT Study
Source: Molecules. 2024 Oct 28;29(21):5086. doi: 10.3390/molecules29215086 (PMC11547787; doi:10.3390/molecules29215086)
Supplement: Supplementary file 1 [file molecules-29-05086-s001.zip › molecules-3239450-supplementary.pdf]

Table S1 The structure of $MoTe_2$, CO, CH_3CHO, and C_6H_6 gases.

Structure	This study (DFT)	Reference (Experiment)	References
$MoTe_2$	Mo-Te: 2.758	Mo-Te: 2.65	[1]
CO	C-O: 1.099	C-O: 1.128	[2]
CH_3CHO	C-H: 1.217<br>C-C: 1.509<br>C-O: 1.217	C-H: 1.09<br>C-C: 1.54<br>C-O: 1.20	[3]
C_6H_6	C-C: 1.399<br>C-H: 1.091	C-C: 1.39<br>C-H: 1.09	[4]

[1] Y. Wang, J. Xiao, H. Zhu, Y. Li, Y. Alsaid, K.Y. Fong, Y. Zhou, S. Wang, W. Shi, Y. Wang, A. Zettl, E.J. Reed, X. Zhang, Structural phase transition in monolayer MoTe2 driven by electrostatic doping, Nature, 550 (2017) 487-491.

[2] W. Xue, Z.-C. Wang, S.-G. He, Y. Xie, E.R. Bernstein, Experimental and Theoretical Study of the Reactions between Small Neutral Iron Oxide Clusters and Carbon Monoxide, Journal of the American Chemical Society, 130 (2008) 15879-15888.

[3] D.P. Stevenson, H.D. Burnham, V. Schomaker, The Molecular Structure of Acetaldehyde, Journal of the American Chemical Society, 61 (1939) 2922-2925.

[4] P. Baltzer, L. Karlsson, B. Wannberg, G. Öhrwall, D.M.P. Holland, M.A. MacDonald, M.A. Hayes, W. von Niessen, An experimental and theoretical study of the valence shell photoelectron spectrum of the benzene molecule, Chemical Physics, 224 (1997) 95-119.
